# A System of Practical Medicine by American Authors

**Published:** 1898-03

**Authors:** 


**A System of Practical Medicine by American Authors.** Edited by ALFRED LEE LOOMIS, M.D., and WILLIAM GILMAN THOMPSON, M.D. Vol. I.—*Infectious Diseases.* Pp. 5–11, 17–985. London: Henry Kimpton. 1897.

Dr. Gilman Thompson in the preface to this volume states that he and the late Dr. Alfred Lee Loomis undertook the joint editorship of this system, and that by securing the co-operation of leading American practitioners and teachers they have been able to assign each subject to an author of the highest repute in its special branch. He further adds: "A closing word is due and affectionately rendered to Professor Loomis. He secured the authors, apportioned their subjects, decided the arrangement and classification of the entire work, and discussed with me many important details, so that in completing the editorial duties alone I have been able to carry out the designs which he had already formulated. He was one of the world's great masters of medicine; he leaves it richer for this result of his knowledge of the science and of his contemporaries who could fitly join him in expounding it. His own article on Endocarditis will be read with all the interest attaching to the latest production of a man universally admired for his attainments and respected for his personality."

The work is to be completed in four handsome imperial octavo volumes, containing from 900 to 1000 pages in each volume. The design is to present a thoroughly practical work, with no extended historical statements and discussions. Each author is expected to present the results of his personal experience in combination with the views of other authorities. There will be no general articles on hygiene, bacteriology, pathology, or symptomatology; but these subjects are to be separately presented in connection with each disease. The system is a work of which the American profession may reasonably feel proud; and the publishers may certainly expect that it will be greatly in demand.

The first article is on "Malaria." This is the joint product of Dr. William H. Welch and Dr. William S. Thayer. The former states definitely that the disease is caused by the specific protozoan parasite called Hæmatozoön malariæ. He proceeds to describe the parasitology of the disease, and gives two excellent plates illustrating the various forms found in the quartan, tertian, and æstivo-autumnal fevers; and also illustrating the connection between the onset of each paroxysm and the ripening and sporulation of a generation of parasites, and the setting free of a new brood: Dr. Thayer details all the clinical phenomena under the heading of "Etiology, pathological anatomy, symptoms, diagnosis, prognosis, and treatment." The authors have still to regret that the manner of existence of the parasite outside the body, and the manner in which the infection takes place, are still wholly unknown. But the action of quinine on the malarial parasite has been abundantly studied, and gives encouragement for more success in the future as regards the treatment of other parasitic diseases.

The article on "Typhoid Fever" is written by Dr. J. C. Wilson, of Philadelphia. In his definition of the disease he states that "Eberth's bacillus is present in the lesions," but he does not emphatically make the parasite the central point of the disease. He gives a terse and excellent summary in 64 pages of the leading phenomena of this well-known malady; and we are glad to note that "there is no proof whatever that this disease in the absence of the germ referred to can be generated by the products of decay or decomposition, by sewer exhalations, by tainted food, or by the action of other bacteria." It is pointed out that the "bacillus completely fulfils two of the requirements of the law formulated by Koch in regard to the evidence that a disease is caused by a given micro-organism: it is present in every case of the disease and in such distribution as will explain the specific lesions, and it can be isolated in pure culture. The third requirement of the chain of evidence, that the disease must be reproduced by inoculation of the isolated organism, remains unfulfilled." We regret to find that the author has not found it needful to adopt the Widal test. He states that "the direct diagnosis of the developed disease finds its support in the continuance of the febrile movement and the appearance of abdominal symptoms;" but he does not tell us how to establish the disease when there are no abdominal symptoms. The diazo-reaction of Ehrlich is clearly of more doubtful utility, inasmuch as it occurs "in many other diseases, especially acute miliary tuberculosis, chronic tuberculosis, measles, scarlet fever, malarial fever, and pneumonia." We venture to think that before another edition of this system is called for, the Widal reaction will have shown itself to be the most trustworthy evidence of the presence of typhoid fever: and that the application of the test will materially reduce the list of infectious fevers of obscure nature described in the last article by Dr. Walter B. James.

The article on "Tuberculosis" is the work of Dr. William Osler, of Baltimore. It is defined as "an infectious disease due to the *bacillus tuberculosis*, characterized by the presence of nodular bodies called tubercles (or diffuse infiltrations) which may undergo caseation or sclerosis, and which may finally ulcerate or in some situations become calcified." In an article of 118 pages we have a valuable record of our knowledge of the effects of the tubercle organism on all the principal organs of the body. Dr. Osler remarks that the Adirondack Cottage Sanitarium, now in the twelfth year of its existence, has proved, under the management of Trudeau, a model institution of its kind: that it "is not only an object lesson of the greatest value in showing how much may be done for the relief of tuberculosis in a properly arranged institution, but it also demonstrates how the scientific study of tuberculosis may be carried on."

We have considered only a few of the important articles contained in this attractive volume, but we trust we have said enough to stimulate our readers to make a further acquaintance with it. Amongst many other useful contributions they will find that smallpox has been dealt with by Dr. William M. Welch, who may be taken to express the best informed opinion on the other side of the Atlantic when he states that "if vaccination were generally practised in infancy and repeated as circumstances required, there would be but little need of considering any other means of protection against smallpox." Dr. W. H. Park's article on "Diphtheria" is one to be carefully studied. He comes to the conclusion that the antitoxin is of decided value, but that it must be used early in the disease, and that it is of the greatest use in uncomplicated cases.

In justice to the publisher it is only fair to state that this set, which promises to be of great practical value, will also be an ornament in any practitioner's bookcase.

